# Synthetic Training Data in AI-Driven Quality Inspection: The Significance of Camera, Lighting, and Noise Parameters

**DOI:** 10.3390/s24020649

**Published:** 2024-01-19

**Authors:** Dominik Schraml, Gunther Notni

**Affiliations:** 1 Group for Quality Assurance and Industrial Image Processing, Ilmenau University of Technology, 98639 Ilmenau, Germany; gunther.notni@tu-ilmenau.de; 2 Steinbeis Qualitätssicherung und Bildverarbeitung GmbH, 98693 Ilmenau, Germany

**Keywords:** synthetic data, rendering parameter, AI inspection, quality control, defect detection, blender

## Abstract

Industrial-quality inspections, particularly those leveraging AI, require significant amounts of training data. In fields like injection molding, producing a multitude of defective parts for such data poses environmental and financial challenges. Synthetic training data emerge as a potential solution to address these concerns. Although the creation of realistic synthetic 2D images from 3D models of injection-molded parts involves numerous rendering parameters, the current literature on the generation and application of synthetic data in industrial-quality inspection scarcely addresses the impact of these parameters on AI efficacy. In this study, we delve into some of these key parameters, such as camera position, lighting, and computational noise, to gauge their effect on AI performance. By utilizing Blender software, we procedurally introduced the “flash” defect on a 3D model sourced from a CAD file of an injection-molded part. Subsequently, with Blender’s Cycles rendering engine, we produced datasets for each parameter variation. These datasets were then used to train a pre-trained EfficientNet-V2 for the binary classification of the “flash” defect. Our results indicate that while noise is less critical, using a range of noise levels in training can benefit model adaptability and efficiency. Variability in camera positioning and lighting conditions was found to be more significant, enhancing model performance even when real-world conditions mirror the controlled synthetic environment. These findings suggest that incorporating diverse lighting and camera dynamics is beneficial for AI applications, regardless of the consistency in real-world operational settings.

## 1. Introduction

Quality assurance in manufacturing, particularly in injection molding, remains a challenge due to a variety of error types stemming from machine parameters, environmental influences, and batch inconsistencies. As these errors can be expensive to produce in real-world settings, synthetic training data offer a compelling solution for machine learning models tasked with defect detection. The use of synthetic data for deep learning has been expanding in various fields in recent years, yet the impact of rendering parameters on the quality of this data and the subsequent performance of AI models is not well understood.

Recent years have seen an increasing number of publications utilizing synthetic training image data for diverse applications. These range from medical domains, such as Schenkenfelder et al.’s [1] generation of virtual images for burn wound detection on human skin, to civil infrastructure inspection, such as Howells et al.’s [2] work that can be used in assessing earthquake damage, and even space technology, where Viggh et al. [3] used Unity to create synthetic data for spacecraft component detection. In industrial contexts, synthetic data have been employed for tasks including segmenting car parts in the automotive industry [4], training robots for bin-picking chicken fillets in the food industry [5], and detecting surface defects on machine parts [6,7,8]. The generation of the synthetic images from 3D geometry is often carried out by using Unity [3,4], and sometimes custom solutions are put into place [7,9], whereas Blender is utilized in the majority of the studies [1,2,5,6,10,11,12,13,14,15,16,17,18,19].

A common approach, exemplified by Schmedemann et al. [17], is the randomization of numerous parameters, including lighting, camera position, texture, and the defect itself. This is based on the assumption that a greater extent of domain randomization leads to enhanced domain adaptation and, thus, improved model performance for real images. While it’s common to alter at least some of the rendering parameters, particularly focusing on camera position and lighting, beyond this assumption, most studies do not delve deeper into the specific impacts of these variations [1,2,3,4,5,6,7,10,12,13,14,15,17,18,19].

Only a few studies have addressed the intricate details of render parameters. Ruediger-Flore et al. [16] explored the impact of realistic material properties and background settings, examining objects rendered at varying levels of detail, from “plain rendered CAD” to “full scene”. Their findings highlight that increasing scene realism significantly boosts classification performance, especially when the proper material properties are added. They also found that mixing different levels of realism and expanding the volume of training data further improves performance. However, their analysis did not extend to other critical parameters, such as lighting and camera position. In a different vein, Zhang et al. [20] compared various illumination models, discovering that the choice of illumination model substantially affects network performance. Yet, their study did not investigate whether variation in lighting is better than using any single illumination model, nor did it determine the characteristics required for one lighting model to excel over another.

This study aims to fill the existing knowledge gap by utilizing the Cycles rendering engine to procedurally generate a specific type of defect (flash) on 3D models of injection-molded parts. With this setup for generating synthetic training data, we focus specifically on empirically evaluating the influence of key rendering parameters—lighting, camera position, and noise—on the efficacy of an AI model for quality assessment. Guiding our research are the following key questions:

1. How does variation in lighting affect the accuracy and generalization capabilities of an AI model trained with synthetic data, as compared to using a static light source?
2. In what ways does changing the camera position impact the accuracy and generalization capabilities of an AI model trained with synthetic data, as opposed to a static camera position?
3. How does the comparison of high versus low noise levels, inherent to the ray tracing process in rendering, affect the accuracy and generalization capabilities of an AI model trained with synthetic data?

The remainder of this paper is structured to explore our methodology, present the experimental results, discuss these results, and conclude with insights aimed at informing future directions in synthetic data generation for AI-driven quality inspection.

## 2. Materials and Methods

In order to create our synthetic 2D image dataset, we use Blender, a versatile piece of open-source 3D graphics software (https://www.blender.org/download/). The integration of the Cycles rendering engine in Blender, which accurately simulates lighting using ray tracing, is essential to the creation of photorealistic images. Blender’s advantages also lie in its rich feature set, which includes 3D modeling and sculpting, texturing, UV mapping, and rendering, as well as its powerful Python API that enables the automation of rendering tasks, which is essential for creating large datasets with variable parameters.

Table 1 outlines the variations in the rendering parameters we examined in Blender, each altered systematically to assess their impact on AI model performance. For instance, for the high noise data, a 0.1 noise threshold in Blender was used, a 10-fold increase compared to the low-noise setting. In the table, the Lighting column contrasts a single light source directly above the object with a high dynamic range imaging (HDRI) background for varied lighting. The Camera Position column compares a fixed camera directly above the object to a camera in random positions within a cube above the object, simulating varying camera positions. The Noise (the noise parameter is linked to ray tracing, where the quantity and path of light rays determine each pixel’s color and brightness in the rendered image) column showcases two scenarios: a high noise scenario with a 0.1 threshold and a low noise scenario with a 0.01 threshold.

### 2.1. Scene Setup

**Figure 1:**
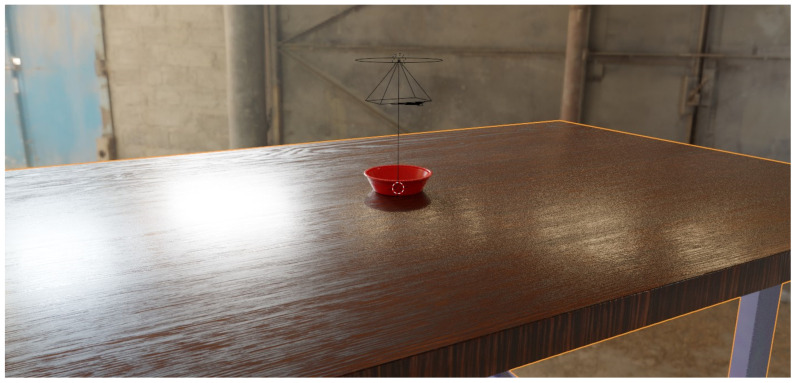
The scene in a rendered preview using ray tracing and a randomly selected HDRI image.

After the 3D modeling of a real injection molded part—a “mortar bowl”—was converted into a .stl geometry, it can be loaded into the blender scene and placed onto a desk geometry. For the material of the desk, we used a freely available asset for a wooden table, meaning the textures for the color, roughness, and normal map are input into the “Principled BSDF” (bidirectional scattering distribution function) shader. For the bowl, we also used the Principled BSDF shader and a Subsurface IOR of 1.46, as well as a Transmission of 0.1 and a Roughness of 0.2 as the properties for plastic material. For the images with variable lighting, a high dynamic range image (HDRI) was used as the background. The scene in Blenders layout view and rendered preview is shown in Figure 1. The reflections of the light sources generated from the HDRI and their physically correct reflection on the tabletop are worth noting, as well as the slight noise in darker areas, such as the right side of the table.

### 2.2. Defect Creation

The sole focus of our simulation is the “flash” defect, a prevalent issue in plastics production and one of the most common defects in injection-molded parts. Flash is characterized by excess material that extends beyond the intended parting line or mold cavity, with its severity ranging from a barely perceptible thread to a wide rim. This defect is not only widespread but is also of significant concern in the industry. It can result from various factors, including improper mold design, inadequate mold maintenance, excessive injection pressure or speed, and material shrinkage during the molding process.

**Figure 2:**
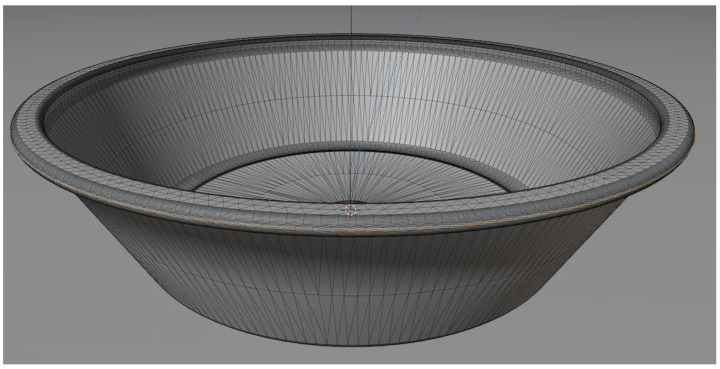
Manipulated vertices highlighted.

The defects on the outer edge of the 3D model were created in such a way that they come as close as possible to those of the real component. In order to do this, the nodes on the outer edge of the 3D object’s mesh were first added to a collection and then saved (see Figure 2). Each time a part with a defect is rendered, the defect creation algorithm iterates over all the points in the collection of the original model’s geometry, randomly selects a subset of vertices, and applies a perturbation to their position, followed by a smoothing process to ensure a natural transition.

The algorithm is parameterized by the ’strength’ of perturbation, the ’perturb_fraction’, which is the fraction of total vertices to be perturbed, and the ’smoothness’, which defines the number of iterations for smoothing the perturbations to create a realistic defect edge. Given a mesh with *N* selected vertices, the algorithm selects a subset of *M* vertices to perturb, where *M* is determined by the perturb fraction:

(1) M=⌈N·perturb_fraction⌉.

Each vertex, $v_{i}$, in this subset is then randomly perturbed by a value, $p_{i}$, within the range [0,strength]. In order to ensure a smooth transition and avoid sharp edges, this perturbation is smoothed over smoothness iterations. During each iteration, the perturbation for a vertex $v_{i}$ is recalculated as the average of its own value and the perturbations of its immediate neighbors, giving the smoothed perturbation $p_{i}^{\mathrm{smooth}}$ as

(2) $$p_{i}^{\mathrm{smooth}}=\frac{p_{i}+\sum_{j\in \mathrm{neighbors}(i)}p_{j}}{\mathrm{number}\;\mathrm{of}\;\mathrm{neighbors}(i)+1}.$$

Finally, the vertices are displaced along their normal vectors by the smoothed perturbation amount, updating the position of each vertex $v_{i}$ to a new position $v_{i}^{\prime }$ by

(3) $$v_{i}^{\prime }=v_{i}+p_{i}^{\mathrm{smooth}}\cdot n_{i}.$$

Adjusting the algorithm’s parameters allows for the simulation of defects that align with the diverse types and severities encountered in actual injection-molded parts. In our experiments, we determined a smoothness setting of 100 to be universally suitable for the type of defects we aimed to replicate. We varied the strength of the perturbation within the range [2.0, 25.0] and the perturb_fraction within [0.01, 0.1]. Both parameters were drawn from a normal distribution with a mean of 0.055 and a standard deviation of 0.025 for the perturb_fraction, and a mean of 13.5 with a standard deviation of 5.75 for the strength. Values falling outside the defined ranges were clamped to the interval’s boundaries.

**Figure 3:**
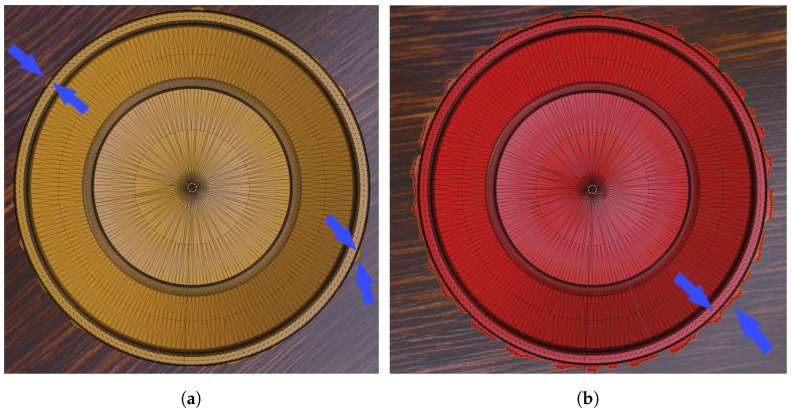
Examples of different defect sizes. (**a**) Median defect strength and fraction; (**b**) maximum defect strength and fraction. Some of the defects at the brim are marked.

The strength parameter determines the scale of the defects, affecting their size and the extent of deformation on the mesh. Conversely, the perturb_fraction sets the occurrence rate of the defects, which translates to the frequency and variability of the defects’ presentation on the mesh surface. In calibrating these parameters, our objective was to replicate defects with a high degree of subtlety. We set the median defect size to be relatively small, and the lower threshold of the strength parameter was selected such that defects at a high perturb_fraction are just barely perceptible, and those at the lower end are virtually invisible to the naked eye without zooming in on the image. This nuanced approach aims to enhance the AI’s sensitivity to minute irregularities, challenging its detection capabilities. Figure 3 depicts defects of median and maximum possible sizes, characterized by excess material on the outer edge (rendered from a top-down camera perspective). The mesh is displayed to better illustrate the variations in the dimensions of these defects.

### 2.3. Render Settings

By randomly selecting one variant of each parameter (as depicted in Table 1), we rendered 10,000 images without defects and 10,000 images with defects, which were generated using the algorithm described in Section 2.2. This resulted in a total dataset of 20,000 images for all variations. Each image was rendered with parameters chosen randomly from those outlined in Table 1, with a 50% probability for each variant. The test dataset, consisting of 4000 images, was rendered separately with the same settings.

Each image was rendered at a resolution of 1920×1080 pixels using the Cycles engine (without denoising) using the GPU. In every rendering cycle, the material color was varied randomly among four options: light blue, dark blue, orange, and red. If the part was to include a defect, it was created by manipulating the object’s geometry with a random size and shape.

With dependence on the randomly selected variant for each parameter, the following render settings were configured via the Python API:

Noise: Adaptive sampling was enabled for both variants. For lower noise levels, the maximum number of samples was set to 500, and the adaptive threshold was set to 0.01. For higher noise scenarios, the maximum samples were reduced to 50, and the threshold was increased to 0.1.

Lighting: Depending on the random selection, either variable lighting was used by selecting an HDRI from 191 available 2 k textures downloaded from Poly Haven [21] or a disk-shaped area light positioned directly above the object with an energy setting of 5000 was used.

Camera: If the top-view camera was selected, Camera Object 1, located directly above the bowl, was used. Otherwise, Camera Object 2 was chosen with its position randomly determined within a cube above the object and oriented towards it.

### 2.4. Preliminary Experiments

**Choice of classification model:** The objective of classification in this research is not to achieve the highest possible accuracy on test or real-world data but rather to assess the comparative impact of different rendering parameters on model performance. The chosen model should not only exhibit good generalization but should also operate efficiently to accommodate the numerous training and inference iterations required by our experiments. We evaluated various models, including different variants of EfficientNet [22] and EfficientNetV2 [23], to determine whether the size of the input images and the complexity of the networks significantly influence performance.

**Image normalization:** The role of image normalization in the training and testing phases was examined by training the model on a subset of 4000 images randomly selected from the entire dataset of 20,000. Three different normalization schemes were applied:

(a) ImageNet with mean = [0.485, 0.456, 0.406], and std = [0.229, 0.224, 0.225];
(b) Rendered dataset mean = [0.3320, 0.2324, 0.2056], and std = [0.1839, 0.1681, 0.1932];
(c) No normalization

**Dataset size**: Rendering a substantial volume of images with accurate light simulation is time-intensive; generating 20,000 images required approximately four and a half days using our equipment. Consequently, to manage the rendering duration—given that seven models were to be trained for our studies—without compromising the integrity of the training data, we evaluated the impact of dataset size on model performance. We considered datasets of 6000 and 8000 images, selected randomly from the full dataset to ensure uniform distribution.

### 2.5. Training EfficientNet

For training the EfficientNetV2-RW-T model, we utilized PyTorch and ensured reproducibility by setting the same seed for all random number generators in torch, numpy, and CUDA. The batch size was determined by the memory capacity of the graphics card, with 24 being the maximum number of samples per batch that could be accommodated. Our dataset was partitioned into an 80/20 split for training and validation. Data loaders were configured with specific transformations for the training and testing datasets (refer to Appendices Appendix A and Appendix B for details). The training process spanned over 40 epochs, utilizing an initial learning rate of $1\times 10^{-3}$.

### 2.6. Obtaining Real Test Data

For all subsequent evaluations (Section 3.2 and Section 3.3), the models were trained and validated according to the methodology described in Section 2.5. The model with the highest validation accuracy after each epoch was selected for further evaluation. We assessed each model’s performance using the synthetic test set and a dataset of real objects photographed under varying environmental conditions. These conditions included placing the object on a wooden workbench and within a specialized inspection device (the inspection device, provided by SQB GmbH, features a back light, top light, and a segmented ring light. It is particularly well-suited for experiments involving variations in lighting and camera parameters, making it an ideal setup for determining optimal image acquisition configurations and for capturing training data) with both light and dark backgrounds. For each setup, we captured images in both well-lit and dimly-lit scenarios, taking two photographs of each object with slight rotations and positional adjustments. Due to the limited availability of real parts with the specified flash defect, only four were available for testing. Conversely, five defect-free parts were used to represent the ‘good’ class. (Note that three of the defective parts are orange, and one is dark blue. For the IO parts, we had one part of each color available).

## 3. Results

### 3.1. Conclusions from Preliminary Experiments

The following results were obtained from preliminary tests to find a valid experimental setup, as described in Section 2.4.

**Choice of classification model**: Initial tests on a subset of 4000 images from our dataset, encompassing all parameter variations, revealed no significant performance disparities attributable to different input sizes (see Appendix A.1). Consequently, with an emphasis on reducing model size and expediting training, we opted for a pretrained version of efficientnetv2_rw_t, which offers a suitable balance between efficiency and predictive capability.

**Figure A1:**
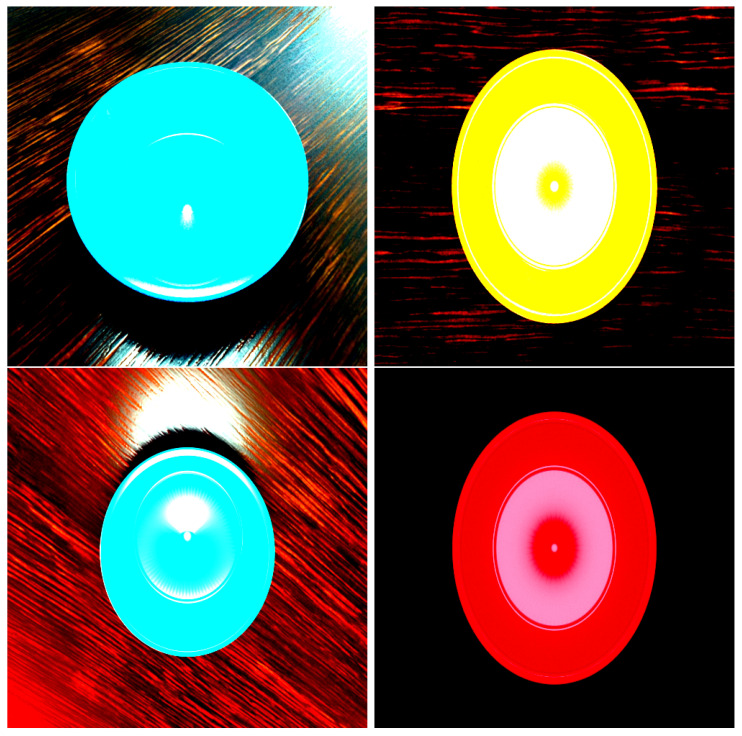
Four examples of rendered images after normalization.

**Image normalization:** Confusion matrices, as shown in Table A4, indicate discernible but modest differences in model performance across the various normalization techniques. Upon visual examination, images after normalization, especially those with darker backgrounds, may lose realism and appear overly dark, as can be seen in Figure A1. In light of these observations and the similar performance metrics of the models tested, we opted against using normalization. This decision also streamlines the testing process.

**Dataset size:** Model performance metrics on the test data are documented in Table A5. The analysis revealed marginal differences among the models, with the model trained on 6000 images (6k) unexpectedly outperforming the one trained on 8000 (8k). Based on these findings, we opted to render a dataset of 6000 images for each of our experiments, with an equal split between the defective and nondefective samples.

### 3.2. Noise

**Figure A2:**
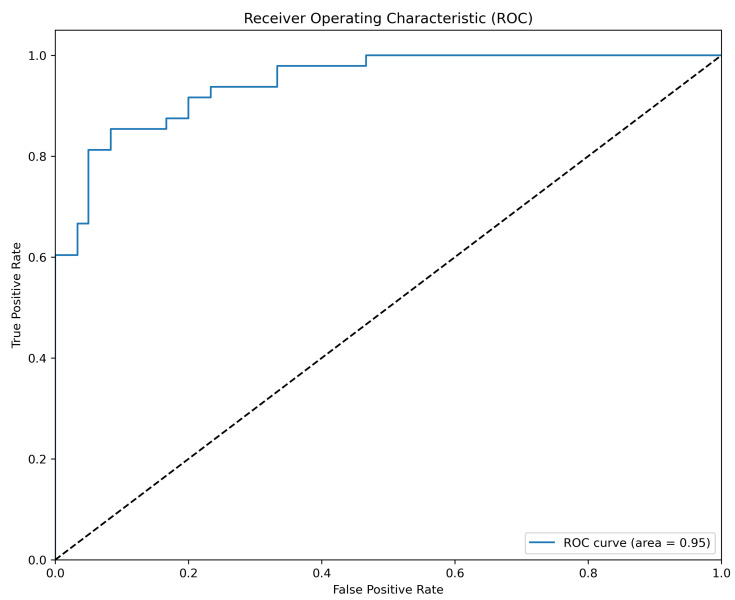
AUC for the high-noise model: 0.9469.

**Figure A3:**
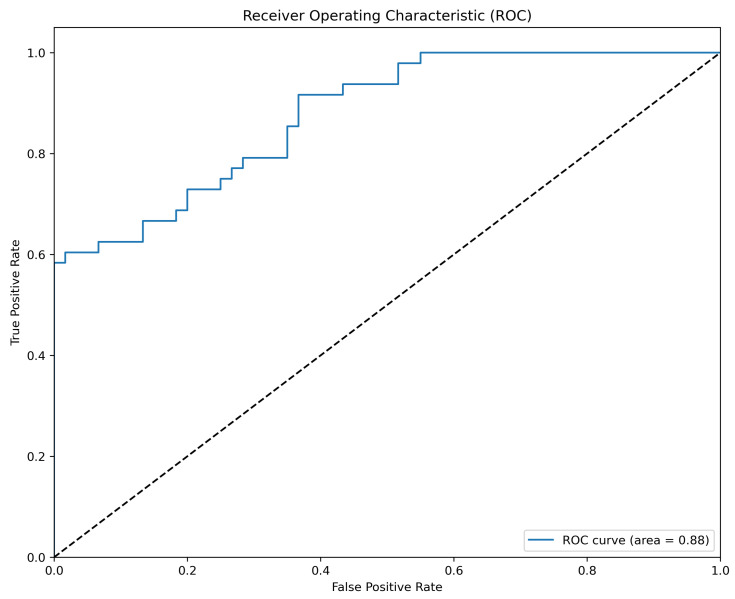
AUC for the low-noise model: 0.8271.

Table 2 and Table 3 present the confusion matrices for the classification results of the models trained on high-noise and low-noise image data, respectively. The left side of each table displays the results on the rendered test dataset, while the right side presents the findings on the real data. Figure A2 and Figure A3 depict the receiver operating characteristic curve (ROC) for the respective noise models when tested against real data. (As a reminder, the area under the receiver operating characteristic curve (AUC-ROC) provides a quantifiable measure of a classifier’s accuracy, plotting the true positive rate (sensitivity) against the false positive rate (1-specificity) at various thresholds. An AUC of 1.0 signifies perfect classification, while an AUC of 0.5 corresponds to the performance of random guessing (diagonal)).

The overall performance of both models on the test datasets is deemed reasonable. The model trained on low-noise images demonstrates a marked reduction in performance on the synthetic test data, suggesting a potential difficulty in generalizing to the high-noise conditions that are also present in the rendered test set. However, when assessed on real-world images, the low-noise model exhibits better performance but still does not quite reach the effectiveness of the high-noise model. It shows comparable proficiency in recognizing defective objects but tends to misclassify a higher number of nondefective parts.

The ROC values provide further insight into the classification abilities of the models: the high-noise model attains an AUC of 0.9469, while the low-noise model reaches an AUC of 0.8271. Both metrics surpass the baseline of random classification, indicating a significant ability to discriminate between classes. Notably, the high-noise model shows distinctly superior performance in accurately classifying real data compared to the low-noise model, underscoring its robustness in practical settings.

### 3.3. Camera Position

**Figure A4:**
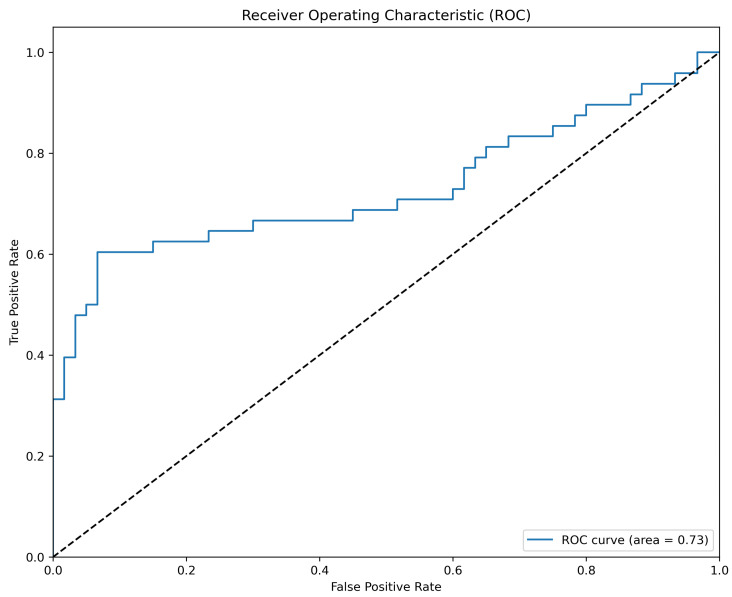
AUC for the top camera model: 0.7295.

**Figure A5:**
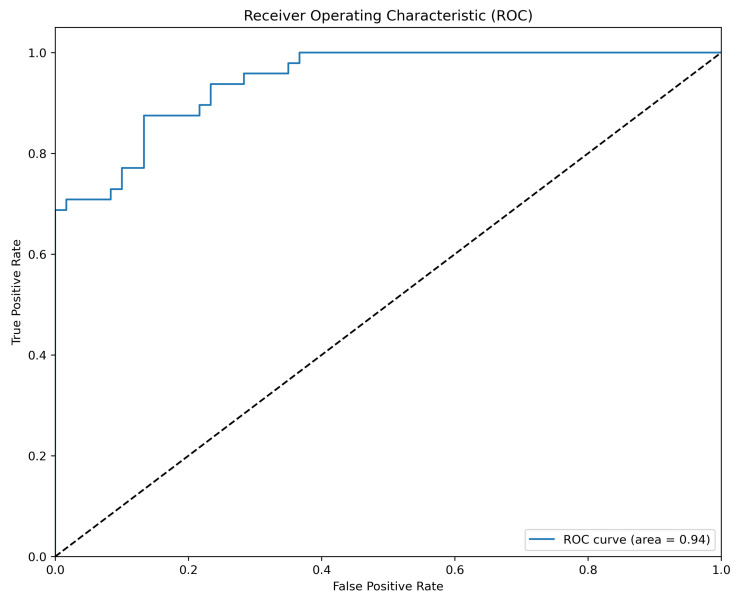
AUC for the variable camera model: 0.9448.

Table 4 and Table 5 show the confusion matrices on the synthetic as well as the real test data. The respective ROC curves are depicted in Figure A4 and Figure A5.

The comparative analysis of model performance, with respect to camera angles, discloses stark differences. The top camera model erroneously classified over half of the nondefective objects within the synthetic test dataset, indicating a significant challenge in generalizing from the training data to varied camera perspectives. In marked contrast, the variable camera model demonstrated consistent and robust performance across the entirety of the synthetic test data. Remarkably, in real-world evaluations, the variable camera model excelled, correctly classifying all nondefective objects—despite the fact that all real images were captured from a top-down perspective. This performance gap is further highlighted in the receiver operating characteristic analysis, where the variable camera model achieved a robust AUC of 0.9448, which is significantly higher than that of the top camera model, the latter nearing the threshold of random classification efficacy.

### 3.4. Lighting

**Figure A6:**
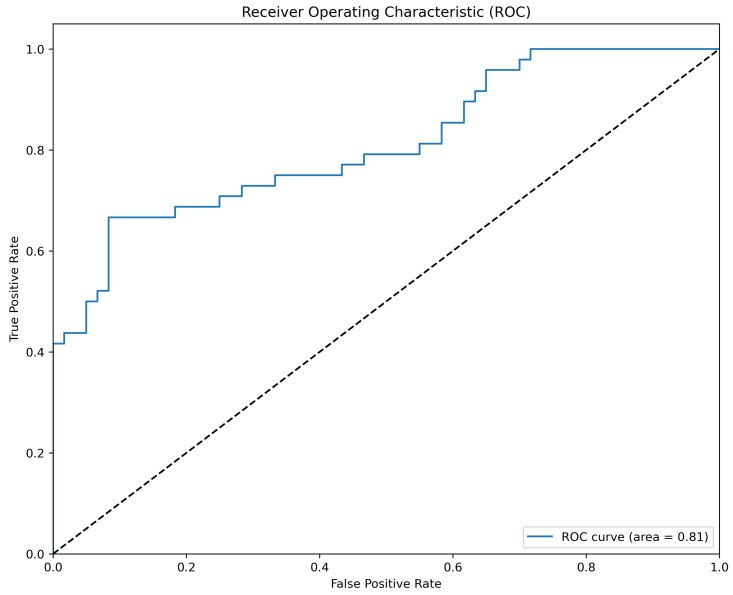
AUC for the top lighting model: 0.8111.

**Figure A7:**
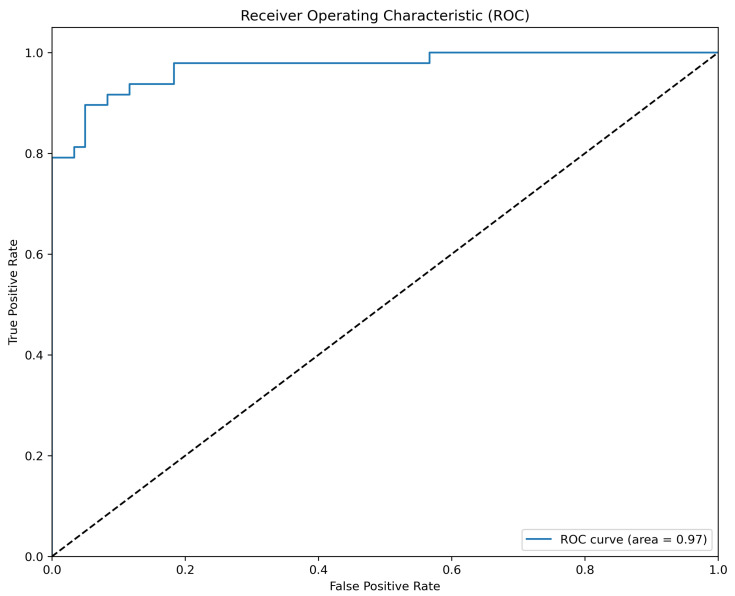
AUC for the variant lighting via HDRI model: 0.9715.

Again, Table 6 and Table 7 show the confusion matrices on the synthetic as well as the real test data. The respective ROC curves are depicted in Figure A6 and Figure A7.

The comparative evaluation between models using HDRI versus top light illumination highlights the subtle yet significant performance variances. On the synthetic tests, the HDRI model outperforms marginally, as it compensates for misclassifying 16 nondefective units by correctly identifying an extra 71 defects. For the real images, while both models accurately classify all nondefective parts, the HDRI model proves superior, detecting 29 out of 48 defects, surpassing the top light’s 17. The AUC metric underlines these outcomes, with the HDRI model achieving a robust AUC of 0.948, showcasing its strong discriminative power, which is in contrast to the top light model’s more modest AUC of 0.7295.

### 3.5. All Variations

**Figure 4:**
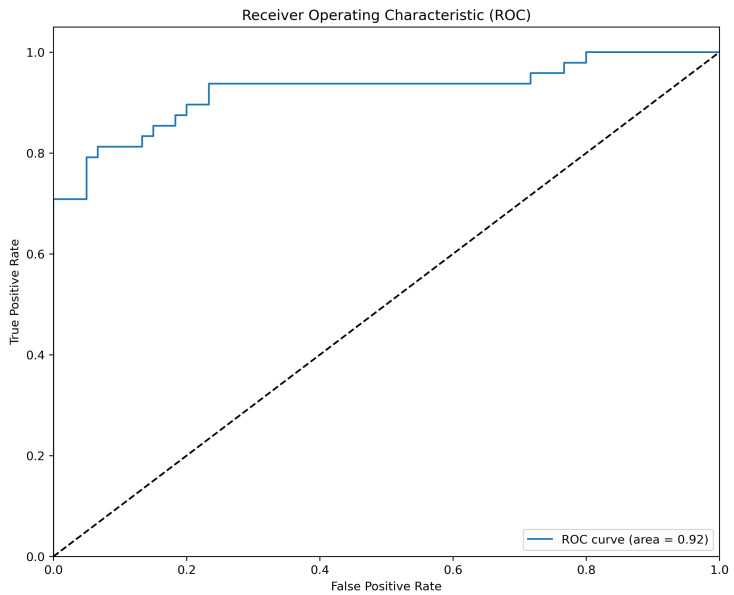
AUC of 0.9233 for the model with all variations using real data.

Upon analyzing the images, it was observed that the model trained with varied rendering parameters predominantly misclassified only those with the smallest flash defects and suboptimal lighting conditions (the metrics are given in Table 8 and Figure 4). In setups with a dark background, every image was correctly classified, and with adequate lighting, all images with larger defects were identified accurately regardless of the background.

**Figure 5:**
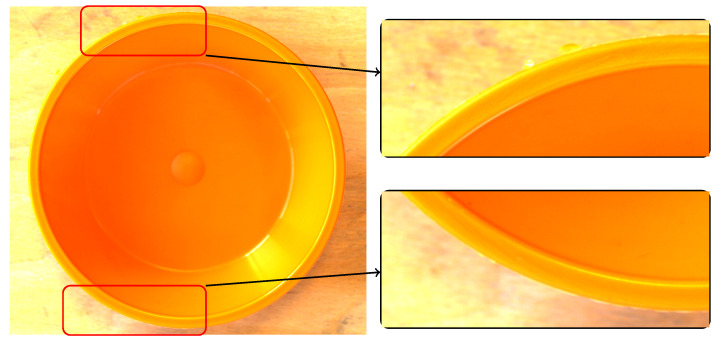
A real part with a small flash that was classified as good.

**Figure 6:**
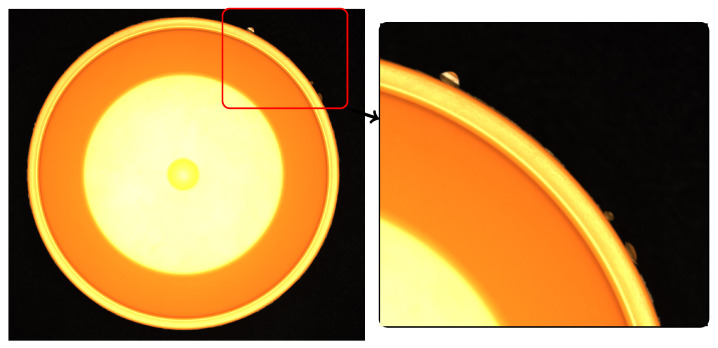
A real part with the smallest flash and darkest background was classified correctly.

Illustrations of these observations include a real image with defects incorrectly classified as nondefective, shown in Figure 5, and an image with the smallest detected defect classified correctly, presented in Figure 6.

Consequently, the experiments culminated, albeit unintentionally, in the creation of an effective classifier, affirming the overall success of the AI training process. This result underscores the model’s adeptness at discerning crucial features for precise defect identification.

## 4. Discussion

In our investigations, we intentionally set the defect parameters to low levels to create subtle defects, resulting in a substantial number of images without recognizable defects. This strategy was not geared towards maximizing classification performance but, rather, was aimed at enabling a meaningful comparative analysis between models trained on varied datasets. During training, we deliberately refrained from using augmentations that could alter image noise or brightness to preserve the purity of the data for assessing the impact of rendering parameters. Universally, the models trained solely on synthetic images demonstrated admirable adaptability when applied to real image data, thus bridging the “reality gap” identified by Tobin et al. [24], and they did so without the need for normalization techniques.

Regarding the **noise** simulation parameter, the high-noise model remained robust when applied to real, lower-noise data (e.g.,Table 2), while the low-noise model struggled to adapt to noisier conditions (e.g., Table 3), suggesting a potential benefit in training with raytracing at lower sampling rates. Similarly, the models with varied **lighting** parameters proved advantageous for classification tasks (e.g., Table 6 and Table 7), indicating the effectiveness of incorporating lighting variation into the training process. The adaptability of models to **camera position** variations also yielded informative outcomes. The models trained with a static top camera perspective performed poorly when faced with deviations in camera angle within the synthetic test set (e.g., Table 4), a trend that was unexpectedly mirrored in real-world scenarios, where the camera positions were consistent, yet the objects were placed only millimeters apart (e.g., Table 4 and Table 5). This leads to our recommendation to include at least minimal camera position variation in rendering synthetic training data for inspection tasks to enhance generalization—even if the actual operational setup involves a fixed camera position.

Our findings also imply the feasibility of prioritizing training with noisy images, which are not only quicker to render via raytracing but also seem to enhance model performance. Including a limited selection of low-noise images might suffice to maintain the model’s ability to generalize.

In our case, the preliminary tests indicated that models without normalization could match or exceed the performance of normalized counterparts on our test data (e.g., Section 3.1). Nonetheless, we advise caution in extrapolating this result to other contexts. In situations where there is significant brightness variation within a dataset, particularly when real and synthetic data are combined, image normalization could be crucial. This area merits further investigation to determine the most effective image data preprocessing techniques for machine learning applications using synthetic data.

## 5. Conclusions

In this work, we addressed the previously unexplored importance of simulation parameters in generating synthetic data for enhancing AI performance. Our approach utilized a binary classification task for defect detection using a single defect type and one 3D model. The study’s findings underscore the significant impact of varying illumination and camera positions on AI performance. Specifically, changing camera position led to a 9.26% (please note that the % sign here and in the following refers to percentage points) increase in model accuracy and a 21.53% improvement in AUC when tested against real-world data. Similarly, employing HDRI lighting instead of top lighting resulted in an 11.11% boost in classification accuracy and a 16.04% increase in AUC. Counterintuitively, we also discovered that training with higher noise not only aids in model generalization but also speeds up data generation, showing a dual advantage with a 5.56% rise in accuracy and an 11.98% enhancement in AUC compared to models trained with lower noise levels.

### 5.1. Limitations

Although this study provides valuable insights into the impact of rendering parameters on AI model performance, it has limitations. Firstly, our investigation into the noise parameter was limited to only two variations, which may not fully capture the spectrum of noise levels encountered in real-world scenarios. Secondly, we did not account for variations in texture in the background, a factor that could significantly influence AI classification accuracy. While focusing solely on binary classification and the “flash” defect type might be seen as a limitation, these choices were made to constrain the scope of the study and manage its complexity.

### 5.2. Practical Implications

Despite these limitations, the practical implications of our research potentially extend far beyond the quality inspection of injection-molded parts. Our findings are pertinent to a broad spectrum of production processes where 3D models are used to algorithmically simulate defects. Tools like Blender or Unity can be leveraged to generate realistic synthetic data, which then serve as training material for AI models tasked with detecting these defects in actual products. Our study demonstrates that manipulating rendering parameters like lighting, camera position, and noise can significantly boost AI model accuracy and generalization. This is vital for those using rendered synthetic data for AI training. Notably, certain parameter variations are more impactful than others, highlighting the importance of a targeted approach in their selection. Effectively prioritizing these key parameters and varying only those that are beneficial can not only streamline the creation of high-quality synthetic training data but also lead to a dataset that is higher in quality, smaller in size, and generated in less time.

### 5.3. Future Research

When looking ahead, several promising research directions emerge. Investigating whether variations in the base or background of synthetic images can further improve model accuracy and adaptability is one of those. Another key area is determining the optimal mix of parameter variations in training data, for instance, understanding if a dataset comprising 80% images with lighting variations outperforms one with a lower proportion of such variations. Furthermore, exploring the benefits of a continuous range in variable parameters, like noise levels, instead of binary high/low levels, might offer more nuanced generalization capabilities, particularly in scenarios involving different sensor types or exposure times. These insights pave the way for future research to optimize synthetic data generation for machine learning applications in quality inspection and beyond.

## Figures and Tables

**Table 1:** Variations of the analyzed cleaning parameters.

Lighting	Camera Position	Noise
One light source directly above the object	Exactly above the object	0.1 Noise threshold
Via the HDRI background	Random position in cube above the object	0.01 Noise threshold

**Table 2:** Confusion matrix for a high-noise model.

	Synth. Data		Real Data	
**Predicted**:	**G**	**NG**	**G**	**NG**
**Actual: G**	1721	279	60	0
**Actual: NG**	182	1818	19	29

**Table 3:** Confusion matrix for a low-noise model.

	Synth. Data		Real Data	
**Predicted**:	**G**	**NG**	**G**	**NG**
**Actual: G**	1483	517	53	7
**Actual: NG**	205	1795	18	30

**Table 4:** Confusion matrix for the top camera model.

	Test Dataset		Real Dataset	
**Predicted**:	**G**	**NG**	**G**	**NG**
**Actual: G**	961	1039	51	9
**Actual: NG**	268	1732	18	30

**Table 5:** Confusion matrix for the variable camera model.

	Test Dataset		Real Dataset	
**Predicted**:	**G**	**NG**	**G**	**NG**
**Actual: G**	1830	170	60	0
**Actual: NG**	241	1759	17	31

**Table 6:** Confusion matrix for the top light model.

	Test Dataset		Real Dataset	
**Predicted**:	**G**	**NG**	**G**	**NG**
**Actual: G**	1850	150	60	0
**Actual: NG**	308	1692	31	17

**Table 7:** Confusion matrix for the HDRI light model.

	Test Dataset		Real Dataset	
**Predicted**:	**G**	**NG**	**G**	**NG**
**Actual: G**	1834	166	60	0
**Actual: NG**	237	1763	19	29

**Table 8:** Confusion matrix for all variations.

	Test Dataset		Real Dataset	
**Predicted**:	**G**	**NG**	**G**	**NG**
**Actual: G**	1840	160	59	1
**Actual: NG**	166	1834	14	34

## Data Availability

We created the synthetic data entirely from the 3D model of the bowl. All rights to this model are owned by Eichsfelder Technik eitech GmbH. https://www.eitech.de/ (accessed on 15 January 2024).

## Appendix A. Experiment Details

### Appendix A.1. Model

As in defect detection tasks, the “not good” (NG) parts are the class of interest we want to primarily identify; the following naming is used:

True Positive (TP): Defective parts correctly classified as NG.

True Negative (TN): Good parts correctly classified as G.

False Positive (FP): Good parts incorrectly classified as NG.

False Negative (FN): Defective parts incorrectly classified as G.

### Appendix A.2. Normalization

The following confusion matrices were the results of training on the 4k images after 40 epochs and inference using the test data.

**Table A1:** Confusion matrices for models trained with different architectures. From left to right: efficientnet-Bb6-ns; efficientnetv2-rw-t; tf_efficientnetv2_m_in21ft1k.

	B6-ns		V2-rw-t		V2_m_in21ft1k	
**Predicted**	**G**	**NG**	**G**	**NG**	**G**	**NG**
**Actual: G**	1823	177	1974	26	1923	77
**Actual: NG**	200	1800	297	1703	273	1727

**Table A2:** Metrics for all models (for NG).

Model	Accuracy	Precision	Recall	F1-Score
efficientnet-Bb6-ns	90.58%	91.05%	**90.00**%	90.52%
efficientnetv2-rw-t	**91.93**%	**98.50**%	85.15%	**91.34**%
tf_efficientnetv2_m_in21ft1k	91.25%	95.73%	86.35%	90.80%

### Appendix A.3. Training Dataset Size

We randomly selected 6000 and 8000 images from the rendered complete dataset of 20,000 images and trained three different models (one on each). The resulting confusion matrices are shown in Table A5.

**Table A3:** Performance metrics for models trained using different normalizations (in %).

Metric	Dataset	ImageNet	None
Precision	92.76%	92.3%	**98.5%**
Accuracy	88.5%	90.7%	**92.0%**
Recall	**91.0**%	89.1%	85.1%
F1-Score	90.6%	90.7%	**91.3%**

**Table A4:** Confusion matrices for models trained using different normalizations.

	Dataset		ImageNet		None	
**Predicted**	**G**	**NG**	**G**	**NG**	**G**	**NG**
**Actual: G**	1862	138	1851	149	1974	26
**Actual: NG**	230	1770	218	1782	297	1703

**Table A5:** Confusion matrices for models trained using different numbers of images.

	6000 Images		8000 Images		20,000 Images	
**Predicted**	**G**	**NG**	**G**	**NG**	**G**	**NG**
**Actual: G**	1910	90	1770	230	1782	218
**Actual: NG**	185	1815	164	1836	143	1857

## Appendix C. Code

**Listing A1.** Train transformation pipeline.
transforms . Compose ( [
transforms . Resize (528) ,
transforms . RandomRotation (degrees = (-30, 30)),
transforms . RandomResizedCrop (size=500, scale=(0.8, 1.0)),
*# both equally likely - 0.5 that neither will occur*
transforms . RandomHorizontalFlip (0.29289322),
transforms . RandomVerticalFlip (0.29289322),
transforms . ToTensor (), ])

**Listing A2.** Validation and test transformation pipeline.
transforms . Compose ([
transforms . Resize (528),
transforms . CenterCrop (size=500),
transforms . ToTensor (), ])
